# Engineering molecular nanoprobes to target early atherosclerosis: Precise diagnostic tools and promising therapeutic carriers

**DOI:** 10.7150/ntno.82654

**Published:** 2023-04-02

**Authors:** Chunfang Zan, Jie An, Zhifang Wu, Sijin Li

**Affiliations:** 1 Department of Nuclear Medicine, First Hospital of Shanxi Medical University, Taiyuan, China; 2 Collaborative Innovation Center for Molecular Imaging of Precision Medicine, Shanxi Medical University, Taiyuan, China

**Keywords:** Atherosclerosis, Early-stage, Nanoprobes, Molecular imaging, Single-modality, Dual-modality, Multi-modality, Precise diagnosis, Therapeutics

## Abstract

Atherosclerosis, an inflammation-driven chronic blood vessel disease, is a major contributor to devastating cardiovascular events, bringing serious social and economic burdens. Currently, non-invasive diagnostic and therapeutic techniques in combination with novel nanosized materials as well as established molecular targets are under active investigation to develop integrated molecular imaging approaches, precisely visualizing and/or even effectively reversing early-stage plaques. Besides, mechanistic investigation in the past decades provides many potent candidates extensively involved in the initiation and progression of atherosclerosis. Recent hotly-studied imaging nanoprobes for detecting early plaques mainly including optical nanoprobes, photoacoustic nanoprobes, magnetic resonance nanoprobes, positron emission tomography nanoprobes, and other dual- and multi-modality imaging nanoprobes, have been proven to be surface functionalized with important molecular targets, which occupy tailored physical and biological properties for atherogenesis. Of note, these engineering nanoprobes provide long blood-pool residence and specific molecular targeting, which allows efficient recognition of early-stage atherosclerotic plaques and thereby function as a novel type of precise diagnostic tools as well as potential therapeutic carriers of anti-atherosclerosis drugs. There have been no available nanoprobes applied in the clinics so far, although many newly emerged nanoprobes, as exemplified by aggregation-induced emission nanoprobes and TiO_2_ nanoprobes, have been tested for cell lines *in vitro* and atherogenic animal models* in vivo*, achieving good experimental effects. Therefore, there is an urgent call to translate these preclinical results for nanoprobes into clinical trials. For this reason, this review aims to give an overview of currently investigated nanoprobes in the context of atherosclerosis, summarize relevant published studies showing applications of different kinds of formulated nanoprobes in early detection and reverse of plaques, discuss recent advances and some limitations thereof, and provide some insights into the development of the new generation of more precise and efficient molecular nanoprobes, with a critical property of specifically targeting early atherosclerosis.

## Introduction

Atherosclerosis, a predominant cause of death and disability worldwide, is well characterized by excessive smouldering inflammation, and lipid metabolism dysfunction in terms of the pathological nature 1-3. On the one hand, abnormal accumulation of apolipoprotein B-containing lipoproteins, mainly low-density lipoproteins (LDLs) in the arterial intima, contributes to early atherogenesis, and following inflammatory responses exacerbate plaque progression over decades, which can lead to the sudden occurrence of fatal cardiovascular events, including plaque rupture, myocardial infarction, stroke, and even sudden death 4-7. On the other hand, advanced plaques are very hard to reverse, and no efficient therapies have been recognized by clinicians so far 8-10. Therefore, monitoring early atherosclerotic progression timely is extremely crucial to reduce these adverse events for patients with cardiovascular diseases.

In fact, there are quite many clinical techniques applied for atherosclerotic plaque monitoring, as exemplified by computed tomography angiography (CTA), contrast-enhanced cardiac computed tomography (CT), intravascular ultrasound (IVUS), etc. 11-13. In contrast to invasive imaging methods, non-invasive imaging techniques for atherosclerotic plaque detection with undoubted advantages, such as noninvasiveness, precision, high spatiotemporal resolution, and low toxicity, have taken a chief place 14, 15. So far, the main non-invasive imaging approaches for patients include ultrasound, X-ray, CT, and magnetic resonance imaging (MRI), having different uses 16-18. Of interest, vascular calcification, a hallmark of atherosclerosis, can be detected by CT, and lipid-rich necrotic core and hemorrhage can be additionally captured by MRI with high sensitivity 19, 20. However, these conventional imaging approaches are limited to the identification of advanced plaques. In other words, it still lacks an effective technique to specifically recognize early-stage plaques, which can be reversed by drug intervention and/or other preventive measures 21, 22. In this regard, developing specific and precise detection methods on the basis of current imaging systems for early-stage plaques is an extremely urgent need. To do so, engineering molecular probes have been emerging and becoming popular to visualize plaque-specific molecules involved in early atherogenesis.

Among various molecular probes, nanoprobes, as a novel type of imaging probes and ultrasmall biosensors, have been shown to contribute to the early diagnosis of multiple diseases, such as Alzheimer's disease 23, and most cancers 24-26. In addition to neurological diseases and cancers, nanoprobes also facilitate the imaging of atheromatous changes through formulating with intracellular and extracellular biomolecules, additionally providing longer blood-pool residence and more specific molecular targeting 27-29. Based on this, the application of nanoprobes in combination with other imaging techniques, as exemplified by ultrasound and MRI, can together achieve more precise imaging effects of the plaque morphology and even molecular/cellular signatures of the atheroma. On the one hand, formulated nanoprobes, have been utilized in the diagnosis of atherosclerosis by combining nuclides, fluorophores, and receptors for the identification of plaques in the field of cardiac nuclear medicine. On the other hand, nanoparticle-mediated combination therapy of atherosclerosis can be realized by introducing therapeutic lipid-lowering or anti-inflammation drugs into the nanoprobes, as a kind of drug delivery system. Of note, formulated nanoprobes can provide both the location information and the expression levels of disease-associated signature biomolecules *in vivo*, eventually leading to early diagnosis of atherosclerosis and other cardiovascular diseases, improved treatment strategies, and accurate assessment of treating efficacy.

Based on this background information, this review aims to give a comprehensive overview of currently-studied nanoprobes in the context of atherosclerosis, summarize published experimental studies showing detective and therapeutic effects of different kinds of engineering nanoprobes on early plaques, go through the contribution of some molecular targets to adding specificity of nanoprobes and discuss current progress and some limitations thereof. Through looking into four common types of single-modality nanoprobes, some dual- and multi-model nanoprobes, and potent molecular targets for early-stage plaque detection and reverse, such as inflammatory mediators and lipid metabolism-related factors, some insights have been gained into the design of more precise and efficient nanoprobes for the recognization of plaque-specific molecules, to achieve early diagnosis and prevention of atherosclerosis eventually.

## General overview of theranostic applications of engineering nanoprobes in atherosclerosis

With regard to the application of nanoprobes in targeting plaques, we would like to start with the contribution of molecular imaging in this context. As a non-invasive strategy, molecular imaging mainly targets specific molecules on the tissue and cell levels, and especially shows their changes in the pathological states, in order to have an in-depth understanding of the disease mechanisms, develop new pharmacological targets, and provide novel drug candidates 30, 31. Given the fact that it is an interdisciplinary subject, molecular imaging indeed involves many different subjects including medicine, radiology, biology, materials science, mathematics, and chemistry 32-34. In terms of atherosclerosis, molecular imaging has the great potential to reveal deeper insights into cardiovascular inflammation and how it evolves over time 35-38. In addition, the successful application of molecular imaging requires not only advanced imaging equipment, such as CT, MRI, and positron emission tomography (PET), but also the synthesis of safe contrast agents 26, 39, 40. Of course, efficient imaging probes, as exemplified by formulated nanoprobes, are extremely important as well. Therefore, considering the potential of nanoprobes in the early detection and diagnosis of atherosclerosis as well as the necessity of targeting early plaques, it is preferable for researchers to develop more sensitive and specific nanoprobes, by which targeted contrast agents can be formed together for thrombosis and plaque imaging.

Proposed applications of engineering nanoprobes in atherosclerosis are mainly divided into two types. First of all, various preclinical studies have shown that formulated nanoprobes can specifically recognize early-stage plaques through targeting plaque-specific molecules. Detection of nanoprobes can be accomplished by a variety of methods. Moreover, the successful imaging of monocytes, macrophages, foam cells, and other plaque components, has enabled these nanoprobes to become promising in realizing the visualization of plaques, especially in the early stage of atherosclerosis 41. Secondly, nanoprobes can act as efficient carriers for anti-inflammation and anti-lipid metabolism drug delivery, assisting in achieving therapeutic purposes 41, 42. In this sense, organic nanoparticles are classical examples to provide both platforms for improved detection (molecular imaging) and more efficacious treatment (drug delivery) of atherosclerosis, owing to their intrinsic physical properties, i.e. superior biocompatibility, and drug-loading capacity 41, 43, 44. To sum up, these engineering nanoprobes can function as potential carriers for both imaging and therapeutic agents for atherosclerotic plaques, in turn extending their traditional clinical applications in this field.

More interestingly, new dual-mode imaging formed by the fusion of multiple ones, such as optical/MR dual-model imaging, optical/ultrasound dual-modality imaging, and photoacoustic/ultrasound imaging and others, has attracted wide attention and tested for atherosclerotic animal models 27, 45-47, as single-mode imaging is not enough to collect accurate imaging information. In addition to overcoming the limitations of single-mode imaging, multi-mode imaging can achieve better imaging effects through combining the advantages of various imaging modalities, to realize the early diagnosis of atherosclerosis. Of note, those fabricated multimodal imaging nanoparticles with reactive oxygen species (ROS)-scavenging ability have been reported to provide a new avenue for the diagnosis and treatment of vulnerable plaques, constructing a novel theranostic nanoplatform for atherosclerosis 48. Overall, recent advances in imaging and treatment of atherosclerosis based on various nanoprobes bring a couple of new opportunities in the future. In the next section, theranostic applications of different kinds of engineering nanoprobes in atherosclerosis will be described in detail according to the number of modalities, with the special focus on comparison of different targeting strategy.

### Single-modality imaging nanoprobes for early detection and prevention of plaques

**Figure 1:**
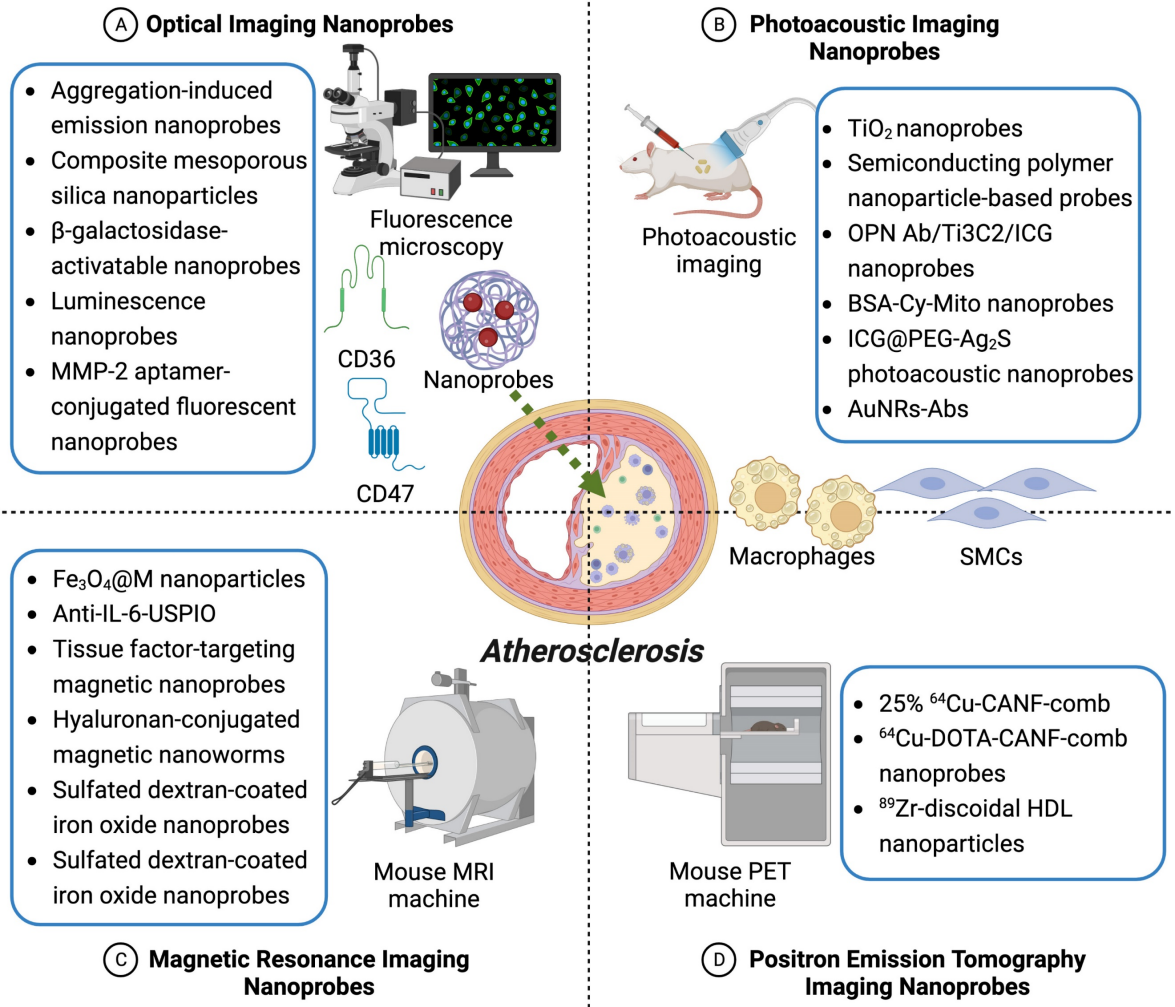
** Single-modality imaging nanoprobes for early detection and prevention of atherosclerotic plaques.** Depicted are four common types of single-modality imaging nanoprobes applied in atherosclerosis research, including optical imaging nanoprobes, photoacoustic imaging nanoprobes, magnetic resonance imaging nanoprobes, and positron emission tomography imaging nanoprobes. Representative nanoprobes in each category are listed in detail here according to current studies.

**Figure 2:**
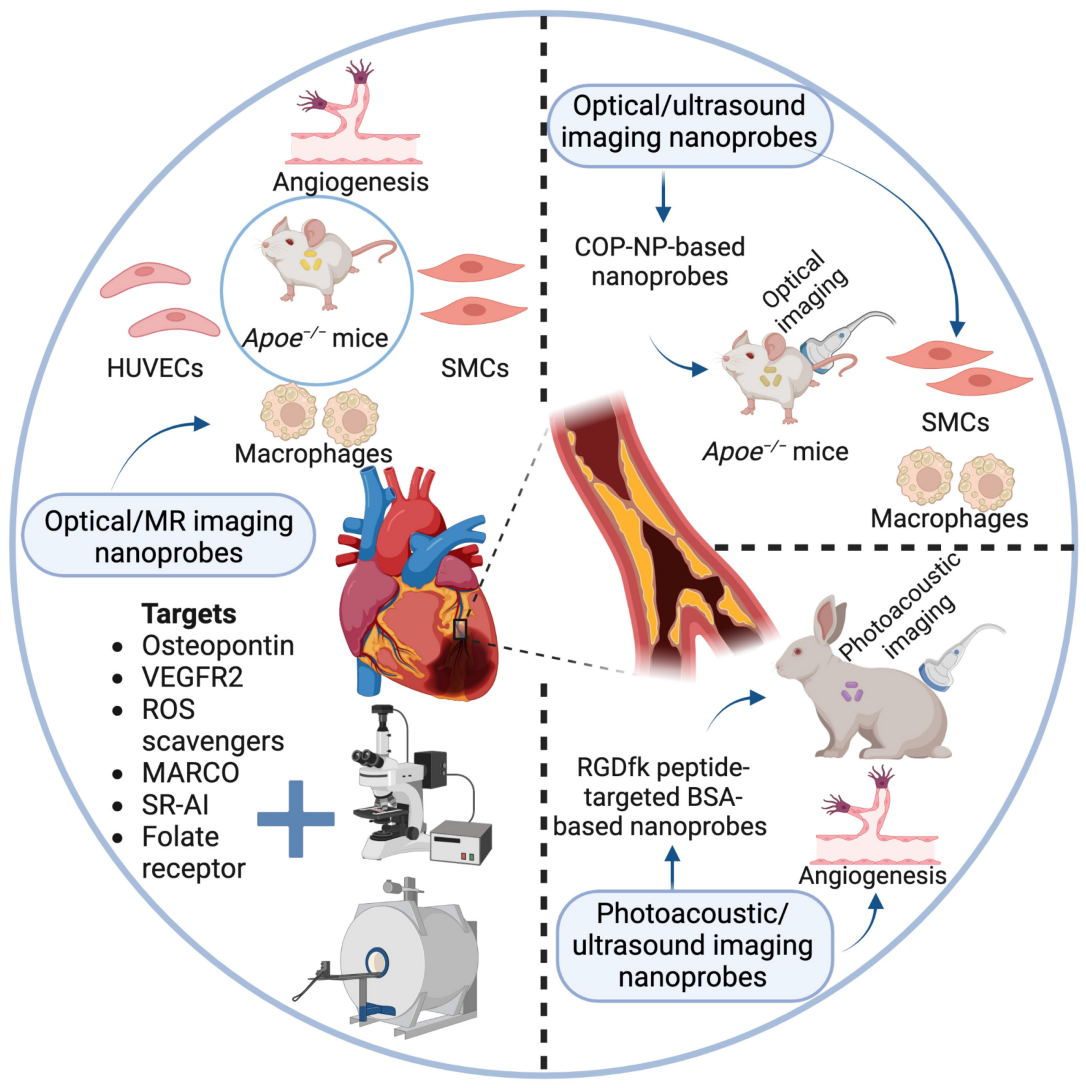
** Optical and photoacoustic-based dual-modality imaging nanoprobes for early detection and prevention of plaques.** Described are three reported types of optical and photoacoustic-based dual-modality imaging nanoprobes applied in current atherosclerotic studies, mainly including optical/MR imaging nanoprobes, optical/ultrasound imaging nanoprobes, and photoacoustic/ ultrasound imaging nanoprobes. Specific molecular targets and biological processes are displayed.

To the best of our knowledge, there has been no systematic review reported for recent advances in formulating nanoprobes in the context of atherosclerosis so far. In this review, comparisons of different kinds of nanoprobes for atherosclerotic plaques have been listed in **Table 1**, covering their major advantages as well as disadvantages. Currently investigated single-modality molecular nanoprobes for plaque detection mainly include optical imaging nanoprobes, photoacoustic imaging nanoprobes, magnetic resonance imaging nanoprobes, and positron emission tomography imaging nanoprobes, which have been summarized in **Table 2** and** Figure 1**, and will be discussed in the following subchapters. Moreover, newly emerging dual-modality imaging nanoprobes will be described in the next section, and summed up in** Table 3** and** Figure 2**. Taken together, these theranostic applications of these single- and/or dual-modality imaging nanoprobes in atherosclerosis, demonstrated by many recent original studies, would give more hope for early diagnosis and prevention of patients with plaques in clinical practice.

### Optical imaging nanoprobes in atherosclerotic research

In the scope of optical imaging, fluorescent materials for labeling, including nano fluorescent probes, have been widely applied to develop optical imaging technology, providing new approaches for early monitoring and treatment of some diseases, such as cancers 49-51. Given the high spatiotemporal resolution as well as the high sensitivity of optical techniques in comparison with other imaging platforms, the application of optical nanoparticles in cardiovascular research has been gradually increasing, as comprehensively reviewed by several recent articles 52-54. As for atherosclerotic studies, optical imaging nanoprobes have multiple advantages for plaque detection, for example, high contrast agent sensitivity, and probe versatility. In addition, optical imaging nanoprobes are very fast and efficient. However, the anatomical information is hard to collect and the quantification is difficult to make, which are the main limitations of optical imaging nanoprobes 26. Even so, there are some original studies to investigate the effects of engineering nanoprobes on detecting and preventing atherosclerotic plaques, highlighting their potential in molecular imaging of atherosclerosis.

Wang and colleagues have recently developed a kind of highly bright aggregation-induced emission (AIE) nanoprobes, which are designed to functionalize with anti-cluster of differentiation (CD) 47 antibodies, to detect early-stage plaques in *Apolipoprotein E*-deficient (*Apoe^-/-^*) mice 28. CD47, as an anti-phagocytic signal for macrophages, has been confirmed to contribute to atherogenesis 55, 56. Of note, CD47-blocking antibodies have been found to restore phagocytosis and meanwhile protect against atherosclerosis in multiple animal models, mechanistically through the regulation of pro-atherosclerotic factor, and tumor necrosis factor (TNF)-α 57, 58. Based on this, these nanoprobes formulated with anti-CD47 antibodies can specifically bind to CD47 overexpressed in atherosclerotic plaques, allowing the efficient recognition of plaques at different stages, especially for the identification of early-stage plaques prior to CT and MRI. Moreover, the clinical use of this kind of fluorescent nanoprobes in targeted imaging of human carotid plaques has been also demonstrated in their study 28. These findings together suggest the potential value of these AIE nanoprobes in monitoring the therapeutic effects of anti-atherosclerosis drugs. In addition to CD47, CD36 is the other target used to develop novel nanoprobes in the context of atherogenesis. Oxidized LDL/CD36 signaling in macrophages has been shown to drive chronic inflammation by mediating dysregulated fatty acid metabolism and oxidative stress from the mitochondria 59, 60. Sun *et al.* have applied CD36-antibody-modified-luminescence nanoprobes for *in situ* imaging of CD36 activation as well as CD36-ox-LDL binding in plaque-associated macrophages with high sensitivity and good stability, which has not been directly visualized in living macrophages by other imaging tools 61. Of interest, the ROS signaling has been observed to enhance the binding of ox-LDL to CD36 in this process, demonstrating new atherogenesis signaling at the cellular level from a different angle 61.

Given the fact that matrix metalloproteinase-2 (MMP-2) contributes to atherogenesis through affecting the functional activities of immune cells, endothelial cells, vascular smooth muscle cells (VSMCs), and platelets 62-64, Han *et al.* have tested an MMP-2-specific aptamer-conjugated fluorescent nanoprobe in *Apoe^-/-^* mice to visualize atherosclerotic plaques 65. They have successfully constructed this kind of fluorescent nanoprobe using a modified DNA SELEX technique and simultaneously achieved good effects through *ex vivo* imaging. It is worth mentioning that this nanoprobe may be available not only for atherosclerotic plaque imaging, but also for gastric cancer tissue visualization. Because MMP-2 aptamer could detect MMP-2 expression in both types of tissues 65. Moreover, considering that atherosclerosis also belongs to aging-associated diseases, β‐galactosidase-activatable nanoprobes, showing good accumulation in arteries, have been developed for *in vivo* imaging of senescent vascular cells in atherosclerotic mice 66. More interestingly, this is the first original study to obtain the successful *in vivo* imaging of senescent cells in pathological vasculatures, although there are several fluorescent probes reported due to the overexpression of senescence-associated β-galactosidases in senescent cells 67, 68. In addition to the accurate identification of plaques, the designed CMSN@SRT@Anti nanoprobes have also shown great potential for targeted therapy of atherosclerotic diseases, due to their excellent biocompatibility, high performance, and superior plaque-targeting ability. After a four-week post-treatment of CMSN@SRT@Anti, persistent fluorescence signals were observed in atherosclerotic lesions, and the aortic plaque area was significantly reduced in *Apoe^-/-^* mice 69. Collectively, the above-mentioned five studies involve different molecular targets of atherosclerosis, and these nanoprobes are conjugated with the same single imaging technique, i.e. optical imaging.

In summary, optical imaging nanoprobes are one type of the most commonly-studied nanoprobes for atherosclerotic plaque detection. So far, CD47, CD36, β‐galactosidase, MMP-2, and osteopontin have been targeted and further validated both *in vivo* and *in vitro*. Even though these formulated nanoprobes are still limited to preclinical exploration, current preliminary data provide a solid basis for their clinical application. In addition to these molecular targets discussed in the above paragraphs, other key players in atherogenesis, such as inflammatory mediators, classical and atypical chemokines, receptors, and lipid metabolism-related factors should be also included in future studies, which would offer more choices for designing more sensitive and feasible nanoprobes under the guidance of advanced imaging systems.

### Photoacoustic imaging nanoprobes in atherosclerotic research

As a non-invasive and non-ionizing biomedical imaging method, photoacoustic imaging (PAI) technology, combining the acoustic resolution and imaging depth of ultrasonography with the sensitivity of optical imaging, has been well developed and applied in the field of atherosclerosis research in the past decade 53, 70, 71. To achieve more specific and high-sensitivity imaging effects of lesional area, PAI nanoprobes have been introduced for plaque detection, with strong light absorption properties, mainly encompassing metal sulfur/selenium/carbide, carbon-based nanoprobes, gold-based nanoprobes, black phosphorus, organic small molecules, for example, indocyanine green (ICG) and melanin, as systematically summarized by two recent review articles 26, 72. Importantly, PAI nanoprobes are beneficial for the differentiation of some specific contents of disease tissues from the control tissues at the molecular level. Of interest, intravascular photoacoustic imaging (IVPA), as a new tool, has shown great potential to visualize both plaque structure and composition, especially for lipid-rich vulnerable plaques 73-75. In this section, we will mainly discuss some relevant original studies investigating newly-designed PAI nanoprobes for atherosclerotic plaque detection and even their therapeutic potential, which have been detailed in **Table 2**.

Wu and coworkers have reported a type of ICG@PEG-Ag_2_S PAI nanoprobes for plaque imaging in *Apoe^-/-^* mice, showing relatively long blood retention as well as selective accumulation in plaques due to the lipophilicity of the C18 chain to the atherosclerotic microenvironment 76. Of interest, ICG@PEG-Ag_2_S PAI nanoprobes have good hemocompatibility and no side effects on the main organs, as shown by hemolysis and coagulation assays. These results together support this fabricated nanoprobe as an available noninvasive imaging tool for atherosclerotic plaques *in vivo* 76. In the same year, Qin and colleagues applied MMP-2-targeted gold nanorods for IVPA of atherosclerotic plaques, encouraging their further development for early diagnosis of atherosclerosis 77. Moreover, two recent experimental studies from Gao *et al.* and Ge *et al.* have developed two kinds of nanoprobes to visualize vulnerable atherosclerotic plaques (VASPs) 78, 79. Gao *et al.* used the self-assembly of bovine serum albumin (BSA), to construct a kind of BSA-Cy-Mito nanoprobe as a GSH/H_2_O_2_ indicator for *in vivo* photoacoustic imaging of redox status in ox-LDL-activated macrophages as well as high fat diet-fed *Apoe^-/-^* mice, in order to evaluate VASP formation with high accuracy. According to their redox states among different types of plaques, BSA-Cy-Mito nanoprobes could specifically distinguish vulnerable and stable plaques, indicating this sensitive redox-responsive PAI nanoprobe may act as a powerful tool for early identification of rupture-prone plaques 78. In a similar vein, Ge *et al*. also focused on features of VASPs using another type of nanoprobes, i.e. formulated osteopontin (OPN) Ab/Ti_3_C_2_/ICG nanoprobes. In addition to the differentiation of VASPs, OPN Ab/Ti3C2/ICG nanoprobes also highlight the importance of OPN in the non-invasively specific imaging of VASPs at the molecular level 79.

Besides, Xie and colleagues have applied a kind of semiconducting polymer nanoparticle-based probe, to specifically identify inflammatory components involved in atherogenesis and further evaluate the inflammation severity by utilizing atherogenic mouse models. Consistent with experimental findings obtained via luminescence nanoprobes formulated by Sun *et al.* and described in the above subchapter, Xie *et al.* also target CD36 and identify the CD36 positive expression as the inflammation level 80. They have demonstrated that the quantification of the PAI signals can reflect the expression of CD36, and the inflammation severity, with good accuracy. At last, TiO_2_-HA-p nanoprobes have been reported as a classical example of metal-based nanoprobes applied in experimental studies for atherosclerosis on the basis of PAI 29. This formulated nanoprobe can specifically target macrophage-derived foam cells, with good photothermal and photodynamic properties as well as excellent biocompatibility. Of special note, black TiO_2_-HA-p nanoprobe-elicited mild phototherapy leads to decreased intracellular lipid levels in foam cells, mechanistically through the regulation of the SREBP2/LDLR pathway as well as ABCA1-mediated cholesterol efflux. This study clearly addresses the therapeutic potential of black TiO_2_-HA-p nanoprobes in atherosclerosis 29.

As demonstrated by the above several studies, PAI nanoprobes have been popularly investigated in the context of atherogenesis. With the advantages of optical imaging and ultrasonography, nanoprobe-based PA imaging is a preferable solution to screen the critical ingredients of atherosclerotic plaques at the molecule level, providing many opportunities for further exploring novel noninvasive imaging techniques of deeper tissues, such as human deeper coronary arteries. Targeting MMP-2, osteopontin, CD36, etc., would further improve the specificity of these PAI nanoprobes, which helps to differentiate advanced vulnerable plaques from early-stage lesions on the basis of the image pattern and the degree of contrast enhancement. However, we have to acknowledge that optical/photoacoustic imaging nanoprobes have been only applied in cell lines and experimental animals due to some limitations of the living biological imaging system. They are often enriched in the liver and difficult to metabolize, which leads to strong background signals and poor imaging quality, preventing them from entering the clinic.

### Magnetic resonance imaging nanoprobes in atherosclerotic research

To date, atherosclerotic plaques and their major components, as exemplified by pro-inflammatory macrophages, have also been extensively characterized by magnetic resonance imaging (MRI), which can generate images with high spatial resolution and excellent soft-tissue contrast 53, 81, 82. Of special interest, superparamagnetic iron oxide nanoparticles (SPIONs), as a type of commonly used MRI contrast agent, show excellent biocompatibility 83-85. However, their specificity is limited and needs to be improved. To tickle this problem, nanoparticle-based imaging contrast agents, in combination with surface-coated molecular targets, such as tissue factor (TF), interleukin (IL)-6, and others, can specifically target the SPIONs to the corresponding epitopes on the macrophage surface, increasing their accumulation in vulnerable plaques, and further improving the accurate detection of atherosclerotic plaques 86-88.

Given the fact that TF is a key proatherogenic factor 89, Wei *et al.* designed EGFP-EGF1-SPIONs as TF-targeted magnetic nanoprobes to precisely and specifically detect TF-expressing cells, such as monocytes/macrophages, endothelial cells, and/or smooth muscle cells in atherosclerotic plaques, which may support to monitor the incidence of early cardiovascular and cerebrovascular events driven by rupturing plaques 86. Of note, the transverse relaxation time (T2) of EGFP-EGF1-SPIONs was remarkably reduced compared with that of SPIONs, indicating that EGFP-EGF1-SPIONs are more suitable negative MRI contrast agents than SPIONs in T2-weighted imaging. Consistent with immunohistochemical quantitative analysis, the TF signal intensity showed a dramatic reduction when plaques progress 86. Huang *et al.* have formulated Fe_3_O_4_@M nanoprobes by coating Fe_3_O_4_ biomimetic nanoparticles with the macrophage membrane, which can effectively target early atherosclerotic lesions by the specific recognition of integrin α4β1 to vascular cell adhesion molecule-1 (VCAM-1) 88. Of interest, the coat of the macrophage membrane on the one hand improves the dispersibility and biosafety of Fe_3_O_4_@M nanoprobes, on the other hand, supports specifically recognizing and binding to VCAM-1 through their highly expressed α4β1 integrin. These features together ensure Fe_3_O_4_@M nanoprobes as a suitable imaging tool for early plaque detection 88.

Moreover, Maiseyeu *et al.* have developed a kind of gadolinium immunonanoparticle-based nanoprobe via targeting inflammation-associated myeloid-related protein (MRP) 8/14, which is an extracellularly secreted protein involved in atherogenesis 90, 91. Chow diet-fed C57BL/6 mice did not show any aortic wall enhancement after anti-MRP-nanoprobe injection, confirming the specificity of these nanoprobes to inflammatory atherosclerotic vessels. Interestingly, MRP-activated macrophages were found to secrete proinflammatory cytokines, and this effect could be reversed by the pretreatment with anti-MRP-nanoprobes, indicating their theranostic potential 91. More recently, Hossaini Nasr and colleagues reported a novel type of hyaluronan-conjugated iron oxide nanoworm (HA-NW) to target CD44-expressing cells 92. CD44 has been found to be overexpressed in plaques, and the CD44-HA axis plays a very important role in atherogenic progression 93, 94. In the meanwhile, HA-NWs display much stronger interactions with CD44-expressing cells in CD44- and HA-dependent manners in comparison with the traditional spherical HA-bearing nanoparticles. Furthermore, successful MRI imaging of plaques by applying HA-NWs has been observed in *Apoe^-/-^* mice *in vivo* 92. Tang and coworkers have developed sulfated dextran-coated iron oxide nanoparticles with specific targeting to macrophages, and ^111^In^3+^ radiolabeled probes were found to bind to the macrophage scavenger receptor A (SR-A) with high-affinity through *in vitro* characterizations. Additionally, higher levels of surface sulfation lead to much higher uptake efficiency by macrophages, as shown by cell uptake studies, revealing a new standard metric for targeted nanomaterials 95. Mo and colleagues have synthesized IL-6-targeted superparamagnetic iron oxide nanoparticles (Anti-IL-6-USPIO), and shown optimized MRI detection of vulnerable plaques in atherosclerotic rabbits 87. Taken together, these studies based on different animal models including mice and rabbits highlight extensive applications of MRI nanoprobes for plaque detection, thereby pointing out a high likelihood of their clinical translation.

To sum up, MRI nanoprobes have shown good prospects for the early diagnosis and treatment of experimental atherosclerosis, even though there are several disadvantages, for example, small molecular weight, short half-life, and high toxicity of some molecular probes. Even so, in comparison to optical/photoacoustic nanoprobes, MRI nanoprobes have a higher possibility to achieve clinical translation, without limitations of equipment. We believe recently-investigated MRI nanoprobes conjugated with different molecular targets would provide more choices for specific recognization and comprehensive evaluation of early-stage plaques.

### Positron emission tomography imaging nanoprobes in atherosclerotic research

Positron emission tomography (PET) is particularly suitable for the non-invasive and quantitative characterization of macrophage-mediated inflammation, vascular calcifications as well as angiogenesis in atherosclerosis owing to the high tissue penetration and superior sensitivity 96-98. However, current PET imaging agents are not targeted. For example, (18)F-fluorodeoxyglucose (^18^F-FDG) lacks specificity for certain cell types, which can be improved by using nanoparticle-based PET imaging agents that has good targeting properties for atherosclerotic plaques.

So far, there are several studies to apply PET imaging nanoprobes in atherosclerotic research, including single-modality as well as dual-modality nanoprobes. In this section, single-model PET imaging nanoprobes are mainly introduced here. Given that angiogenesis is a complex biologic process in atherosclerosis 99, Liu and colleagues constructed a C-type atrial natriuretic factor (CANF)-conjugated nanoprobe, i.e. DOTA-CANF-comb nanoprobe, to detect natriuretic peptide clearance receptor (NPR-C) levels with PET in an animal model with atherosclerosis-like lesions 100. The targeted DOTA-CANF-comb nanoprobe shows significantly higher tracer accumulation in comparison with either the nontargeted control nanoprobe or the CANF peptide tracer, as demonstrated by PET imaging. The following immunohistochemistry confirms the upregulation of NPR-C in the angiogenic lesion, which is colocalized in both endothelial and smooth muscle cells 100. Afterwards, the same research group validated the application of this nanoprobe in atherosclerosis imaging *ex vivo* and *in vivo*, and further addressed its potential for clinical translation 101.

Considering that high-density lipoprotein (HDL) is a natural nanoparticle that interacts with macrophages in atherosclerotic plaques, Pérez-Medina *et al.* developed ^89^Zr-HDL nanoparticles in combination with noninvasive PET imaging, to visualize its accumulation in advanced plaques. Of special interest, this formulating nanoprobe has been validated in different kinds of atherosclerotic animal models, such as *Apoe^-/-^* mice, rabbits, and pigs 102. Woodard and colleagues applied three types of ^64^Cu-CANF-comb nanoprobes to assess the *in vivo* PET imaging of NPR-C, which is expressed on atherosclerotic plaques, and found that the 25% ^64^Cu-CANF-comb shows the best NPR-C targeting specificity as well as sensitivity in *Apoe^-/-^* mice, suggesting the 25% ^64^Cu-CANF-comb as a good PET imaging agent to detect atherosclerosis 103. Therefore, these three studies show various designs of PET imaging nanoprobes in terms of different features of atherosclerosis, i.e. angiogenesis, lipoproteins, and inflammatory macrophages.

### Dual-modality imaging nanoprobes for early detection and prevention of plaques

To date, dual-modality imaging nanoprobes have been becoming popular, as single-mode imaging is not enough to collect accurate imaging information of plaques. Of note, dual- or multi-modality simultaneous imaging techniques facilitate the integration of information on both anatomy and function, and thus have the potential to improve diagnostic and prognostic evaluation for atherosclerosis 104-106. The most common pattern of dual-modality imaging nanoprobes for plaque visualization is optical/MR dual-model imaging nanoprobes, and other patterns such as optical/ultrasound, photoacoustic/ultrasound, PET/MR, and PET/CT dual-modality imaging nanoprobes, as summarized in **Table 3** and **Figure 2** (partially), have also been discussed in this section.

### Optical/MR dual-model imaging nanoprobes in atherosclerotic research

In addition to the application of single optical imaging, the hybrid optical/MR imaging system is also involved in atherosclerotic research. MRI is applied to collect the anatomical information of the diseased blood vessels, and optical fluorescence imaging can further obtain cellular and molecular information due to its high sensitivity 107-110. Therefore, this bimodal imaging can offer a more precise mode for vulnerable plaques, which has been reported to coordinate with TPZ/IR780@HSAeOPN nanoprobes or Fe_3_O_4_ nanoparticle-based probes, and introduced in this subchapter as well 27, 111. Both nanoprobes target osteopontin, which shows a high expression pattern in plaques, and is closely associated with smooth muscle cell proliferation and foamy macrophage formation 112, 113. Based on this rationale, Xu together with colleagues established osteopontin-targeted theranostic nanoprobes, which can precisely regress VASPs through a cascade of synergistic events triggered by local irradiation of lasers under the guidance of fluorescence/MR imaging. This hybrid imaging system on the one hand supports that the osteopontin-targeted TPZ/IR780@HSAeOPN nanoprobes could selectively accumulate in the VASP lesions, on the other hand, enables the precise near-infrared (NIR) laser irradiation to generate massive ROS, which results in efficient plaque ablation and amplified hypoxia within VASPs 27. In a similar vein, Qiao *et al.* have applied osteopontin-targeted probes based on Fe_3_O_4_ nanoparticles to achieve MRI/optical dual-modality imaging of VASPs 111. It is worth noting that Fe_3_O_4_ nanoparticle-based probes have low toxicity, which makes them available in the noninvasive evaluation of early plaques. In their study, these formulated nanoprobes have been found to specifically recognize foamy macrophages through *in vitro* cell experiments, and further visualize vulnerable plaques as demonstrated by *in vivo Apoe^-/-^* mouse model studies 111. The same research team tested the validity of another OPN-specific upconversion luminescent probe (UCNP-anti-OPN) based on the same mechanism 114.

Owing to that macrophages are key components of atherosclerotic plaques, several researchers have developed dual-modality nanoprobes using molecular targets related to macrophage activation, which would be discussed in detail in this section. Yao and colleagues targeted folate receptor (FR)-β, and formulated a dual-modal fluorescent/MRI contrast agent to detect inflammation-associated activated macrophages within carotid plaques 45. The reason why they chose FR-β in this study is that FR-β is a specific marker of macrophage activation, and FR-expressing macrophages kind of represent plaque area 115-117. Additionally, M1 macrophage polarization in plaques has been visualized by optical/MRI dual-modality imaging with MARCO-targeted upconversion luminescence probes, displaying the behavior of M1 phenotype macrophages in *Apoe^-/-^* mice 46. Another studied target associated with macrophage activation is scavenger receptor-AI (SR-AI), which has been conjugated with ultrasmall gold nanoclusters to facilitate dual-modality imaging of vulnerable plaques 118.

In addition to macrophage activation, Fang and coworkers mainly looked into biomarkers associated with the angiogenesis process, and eventually achieved dual-modality imaging of unstable plaques via utilizing VEGFR2-targeted upconversion nanoprobes. Of note, FITC-VRBP1 has been observed to bind to HUVECs with high specificity. Furthermore, the successful optical/MR dual-modality imaging targeting angiogenesis in plaques has been obtained through applying VRBP1-UCNPs, confirming that it is a promising technique to detect unstable plaques in early-stage 119. Unlike these studies, Wang *et al.* developed profilin-targeted magnetic nanoparticles, i.e. PC-NPs, as dual-modality molecular probes for murine atherosclerosis, and revealed their potential in characterizing VSMCs in plaques. Importantly, a good correlation between MRI signals and fluorescence intensities of imaging in* Apoe^-/-^* mice with PC-NPs injection was observed 120. At last, Dai *et al.* submitted that the ROS-scavenging nanoparticles can mediate MR/fluorescence dual-modality imaging tracing of vulnerable plaques. It is worth pointing out that this is the only study to especially address the therapeutic potential of dual-modality nanoprobes in atherosclerosis, mechanistically through the downergulation of inflammation, apoptosis, and foam cell formation 48. Taken together, optical/MR dual-model imaging nanoprobes have been under active investigation, and have gradually shown promising potential in early diagnosis and prevention of atherosclerosis.

### Optical/ultrasound dual-modality imaging nanoprobes in atherosclerotic research

Among various diagnostic imaging techniques, ultrasound imaging has its own advantages for atherosclerotic plaques, for example, real-time monitoring capability, low cost, portability, and high safety 121, 122. Thus, optical/ultrasound dual-modality imaging is a preferable integrated choice in this sense. Li and coworkers characterized osteopontin-targeted nanoparticles, i.e. COP-NPs, and showed that these nanoparticles are accumulated in vulnerable plaques, as demonstrated by both ultrasound and optical imaging. Of note, osteopontin-targeted nanoparticles have been verified as a good contrast agent in molecular imaging of foam cells and smooth muscle cells, which thereby can be an efficient tool to identify vulnerable plaques 123.

### Photoacoustic/ultrasound dual-modality imaging nanoprobes in atherosclerotic research

For many years, an integrated intravascular imaging catheter has been successfully designed and developed, making it possible for the clinical realization of the hybrid intravascular ultrasound/intravascular photoacoustic (IVUS/IVPA) imaging system 124, 125. Based on this novel imaging platform, Lin *et al.* constructed RGDfk peptide-targeted nanoprobes to study angiogenesis in atherosclerotic plaques, not only obtaining functional information of multiple components in plaques, such as neovascularization and lipid core, but also acquiring arterial structural information. In their study, an atherosclerotic rabbit model was utilized, and the imaging effects of photoacoustic/ultrasound dual-modality imaging nanoprobes on plaques were systematically evaluated *in vivo* 47. From a clinical perspective, Yeager and coworkers successfully achieved *ex vivo* coronary artery plaque photoacoustic/ultrasound imaging in a patient undergoing autopsy by using silica-coated gold nanorods as contrast agents 126. Even though there are few studies in this regard, hybrid intravascular molecular imaging technology is promising to provide a reliable platform for the early detection and intervention of atherosclerosis.

### PET/CT dual-modality imaging nanoprobes in atherosclerotic research

Due to its high sensitivity and quantitative diagnosis, the integrated approach of PET/CT has been widely studied in preclinical and clinical atherosclerotic research among various integrated imaging modalities 127-129. In this context, PET/CT imaging nanoprobes are mainly designed to target chemokine receptors, as exemplified by CCR5 which is mostly expressed in monocytes and neutrophils 130-132. Luehmann *et al*. applied ^64^Cu-DOTA-DAPTA-comb and ^64^Cu-vMIP-II-comb nanoprobes in different studies, respectively, and found that plaque progression was in line with increased expression of chemokine receptors as well as elevated PET signals. These results together indicate the potential of PET/CT imaging nanoprobes in detecting plaque progression in C57BL/6 mice and/or *Apoe^-/-^* mice 130, 131. In addition, the latest study from Detering and colleagues showed the application of CCR5-targeted peptide D-ala-peptide T-amide, i.e. DAPTA-comb nanoprobes, in PET/CT imaging of atherosclerotic plaques, and revealed physicochemical properties and targeting efficiency of DAPTA-comb. All three ^64^Cu-DAPTA-comb nanoprobes could visualize lesions by detecting CCR5 expression with high sensitivity and specificity 132. Even though PET/CT imaging nanoprobes show broad prospects, their applications are also challenged, because that PET/CT imaging has some limitations, such as low soft-tissue contrast, and CT-related radiation exposure. For this reason, more PET/MR dual-modality imaging nanoprobes are under active investigation, which will be described emphatically in the following section.

### PET/MR dual-modality imaging nanoprobes in atherosclerotic research

Nowadays, PET/MR dual-modality imaging has been extensively applied to study inflammation in atherosclerosis by mapping key molecular processes as well as functional parameters, as reviewed by Senders *et al.* recently 133. Of note, high soft-tissue contrast can be achieved by PET combined with MR imaging. In clinical practice, Li *et al.* utilized [^68^Ga]Pentixafor PET/MR imaging to evaluate the expression of CXCR4 in human atherosclerotic lesions, highlighting CXCR4 as a surrogate marker for atherosclerosis 134, 135. Similarly, several types of PET/MR dual-modality imaging nanoprobes have been experimentally investigated to improve the molecular diagnosis of atherosclerotic plaques, by targeting inflammatory macrophages, oxidized phospholipids, vascular calcifications, and angiogenesis.

Jarrett and colleagues synthesized ^64^Cu-labeled dextran sulfate-coated iron oxide nanoparticles, and thereby mapped macrophage distribution in lesion area using PET/MR imaging. Then, the enhanced contrast induced by these nanoprobes was observed at sites of vascular inflammation, but not in a normal vessel, in both PET and MR images 136. In a similar vein, Tu *et al*. developed a kind of multifunctional PET/MRI nanoprobe, i.e. ^64^Cu-NOTA-IONP@MMP2c-PEG2K, MMP2cNPs, to evaluate the expression of MMP-2 in macrophage-rich vascular lesions. Interestingly, the excellent plaque-to-normal carotid artery contrast was obtained due to the rapid clearance of MMP2cNPs from the contralateral normal carotid artery. Moreover, iron was accumulated in atherosclerotic plaques, which was colocalized with MMP-2 in macrophages, as confirmed by histological analyses 137. Taken together, the above-mentioned two kinds of nanoprobes are mainly assisting the molecular imaging of inflammatory macrophages in atherogenesis.

In contrast, Su *et al*. mainly looked into angiogenesis in a rabbit atherosclerotic model via using GEBP11 peptide-targeted magnetic iron oxide nanoparticles, showing good imaging properties, high stability, and little cytotoxicity as well as low immunogenicity, in the context of PET/MR dual-modality imaging 138. Afterwards, Pellico and coworkers applied ^68^Ga iron oxide nano-radiomaterials for the targeted bioorthogonal molecular imaging, with their selective accumulation in atherosclerotic plaques in *Apoe^-/-^* mice 139. More recently, Pellico *et al.* reported hydroxyapatite (HAP)-multitag as a PET contrast nano-tracer for the characterization of vascular calcifications in plaques, and revealed that HAP-multitag can support to achieve the early detection of plaques in 16-week-old *Apoe^-/-^* mice. Importantly, these imaging probes are capable of providing simultaneous signals in both modalities, i.e. PET/MRI 97. In summary, PET/MR dual-modality imaging allows for non-invasive studies of atherosclerotic progression, and relevant molecular mechanisms as well.

### Multi-modality imaging nanoprobes for early detection of plaques

Not only high-sensitivity imaging techniques but also specific targeting markers are needed for an ideal imaging platform to identify atherosclerotic plaques precisely and specifically. Therefore, developing multi-modality imaging nanoprobes is an appealing trend in the future, although there might be some technical challenges in constructing nanomaterials with tailored physical and biological properties. In fact, various multi-modality imaging has been applied to study tumorigenesis 140, 141, while few studies are available for atherosclerotic research. Dating back to 2008, Nahrendorf and colleagues performed multi-model imaging of macrophages in plaques by using a tri-modality reporter for PET, MRI, and fluorescence imaging, i.e. ^64^Cu-TNP, and observed a pronounced correlation between PET signals and CD68 expression. Of interest, they addressed the diagnostic capability of multi-modality nanoprobes in atherosclerosis and their clinical translatability as well 142. Recently, Tong *et al.* developed a novel multimodal imaging agent, 5-HT-Fe_3_O_4_-Cy7 nanoparticles, for MRI, CT, and fluorescence imaging of vulnerable plaques by detecting active myeloperoxidase, displaying high sensitivity and specificity. The severity of inflammation and the activity of myeloperoxidase could be evaluated according to the accumulation of these nanoprobes in plaques 143. With more relevant studies emerging, the potential of multi-modality imaging nanoprobes will be revealed, especially for early-stage plaques.

### Molecular targets to formulate nanoprobes for atherosclerosis imaging

Considering the importance of molecular targets in determining the specificity of nanoprobes for plaque visualization, we would discuss currently-applied targets and some other potent candidates in this section, separately. In the meanwhile, relevant details and comparison of several well-investigated molecular targets, encompassing VCAM-1, OPN, MMP-2, CD47, CD44, MARCO, and VEGFR-2, etc., have been summarized in **Table 4** as well as** Figure 2**.

Current plaque-relevant molecular targets that have been chosen for the design of nanoprobes, mainly include aptamers, peptides, antibodies, and others. In general, antibodies have high specificity and affinity, whereas high immunogenicity and poor stability. Additionally, high cost is also a disadvantage limiting antibodies' application. In contrast, peptides need relatively low production costs, with high specificity and safety but limited stability 144. More recently, in terms of the high immunogenicity of antibodies as well as the low stability of peptides, aptamers with a couple of advantages offer a better type of ligands to construct new nanoprobes 145. Thus, it is essential to choose the proper ligand pattern when designing nanoprobes for plaque imaging, although current studies have tested various ligands and made some progress to a certain extent.

On the other hand, as shown in **Table 4**, these targets are closely associated with different biological processes in atherogenesis, for example, inflammation, angiogenesis, vascular calcifications, and lipoprotein synthesis as well as clearance 28, 61, 77, 93, 102, 119. Among them, inflammatory factors are the most common targets, such as IL-6, MARCO, CD36, CD47, and others. Nowadays, special attention is called for many other candidates which have not been tested yet but simultaneously involved in several pathological processes, as exemplified by some classical chemokines and atypical chemokines. CXCR4, as a known receptor for CXCL12 and macrophage migration inhibitory factors (MIFs), participates in atherogenic inflammation and lipid metabolism, which may support it as a promising candidate for the design of more specific nanoprobes to visualize plaques 146. Therefore, exploring specific and selective molecular targets is a key step for the generation of novel nanoprobes. Based on this, it makes sense to further evaluate the imaging effects of plaques. In the future, more mechanistic investigation is still needed to discover new molecular targets of atherogenesis, supporting the construction of novel nanoprobes in this field.

## Concluding remarks and future perspectives

In recent years, molecular imaging techniques that enable early monitoring of atherosclerotic plaques before clinical manifestation is an active area of research. Various single-, dual- as well as multi-modality imaging nanoprobes have been investigated and show promising prospects. However, optical/photoacoustic imaging nanoprobes have been only applied in cell lines *in vitro* and experimental animals *in vivo,* due to some limitations of the living biological imaging system, as exemplified by fluorescent nanoprobes. Especially, they are often enriched in the liver and difficult to metabolize, which leads to strong background signals and poor imaging quality. Although these shortcomings prevent their clinical application, they can still be utilized to study the pathological mechanisms of atherosclerosis, and evaluate the drug efficacy. In contrast to optical/photoacoustic nanoprobes, MR/PET nanoprobes have a higher likelihood to achieve preferable clinical translation once their specificity and selectivity are improved, without limitations of equipment.

In this regard, it is worth noting that there are several potential factors preventing these nanoprobes from entering the clinic, as implied by many original studies. First of all, targeting efficiency is a key issue. Owing to the limited specificity of current nanoprobes in detecting plaques, target-specific ligands are extremely needed to further improve the accuracy of the molecular diagnosis of early atherosclerosis. Nowadays, researchers have developed various types of nanoprobes coated with well-studied plaque-specific molecules, exerting tailored physical and biological properties. They on the one hand are beneficial to specific recognization of early-stage plaques, on the other hand, give additional insights into the molecular basis for atherogenesis. However, the presence of the same receptor in different cells still reduces the targeting capacity of the molecular probes, in turn limiting its clinical application and drawing more attention from researchers. Secondly, we have to consider the metabolic issues of nanomaterials when it comes to their application in patients. Recently, metabolizable near-infrared-II nanoprobes seem to be a better choice to overcome this drawback. Thirdly, the complexity and the required dosage of these nanoprobes for imaging need to be optimized to achieve the medical purpose, which is also one obvious limitation. Thus, these key factors should be taken into account when designing novel nanoprobes in the context of atherosclerosis.

In addition, dual- and multi-mode imaging combining the advantages of multiple single-mode imaging techniques become more popular, as single-modality imaging is not enough to provide accurate imaging information. For this reason, developing dual- and multi-modality nanoprobes and/or combining multiple molecular targets in one type of nanoprobe is the common trend in this field. Furthermore, rigorous validation of the signal origin and the probe fate would be guaranteed by the multi-modality capabilities of these nanomaterials. Undoubtedly, specific targets, metabolizable nanomaterials, and advanced equipment, are together to support the precise visualization of plaques. Of interest, cell membrane-coated nanotechnology has emerged as a promising therapeutic platform, which may facilitate the targeted delivery of anti-atherosclerosis drugs. In the future, multi-modality nanoprobes with multiple targets would strongly contribute to accurate diagnosis and efficient treatment of atherosclerosis, although their complexity may bring some technical difficulties.

In summary, this paper reviews different types of formulated nanoprobes applied for early detection and reverse of plaques, and especially highlights recent advances, many challenges as well as opportunities. Now clearly, with the development of metabolizable nanomaterials and the presence of more specific targets, the clinical value of nanoprobes with the critical property of specifically targeting early atherosclerosis has been increasing. Of special note, the new generation of more precise and efficient molecular nanoprobes also needs the close collaboration of cardiologists, chemists, clinical radiologists, radiobiologists, statisticians, molecular biologists, and immunologists. Taken all together, engineering nanoprobes, serving as precise diagnostic tools and promising therapeutic carriers, would provide more preferable choices for early diagnosis and prevention of atherosclerosis.

## Figures and Tables

**Table 1:** Comparisons of four major types of single-modality nanoprobes for plaque detection

Types	Detection	Examples	Main advantage	Main disadvantage
Optical imaging nanoprobes	Fluorescence	AIE nanoprobes; luminescence nanoprobes	High contrast agent sensitivity	Low tissue penetration
Photoacoustic imaging nanoprobes	Ultrasonic waves	TiO_2_ nanoprobes	High contrast and deep tissue penetration	Toxicity
MR imaging nanoprobes	Magnetic field radio waves	Tissue factor-targeting magnetic nanoprobes	High spatial resolution	Difficult to quantify
PET imaging nanoprobes	γ-ray	DOTA-CANF-comb nanoprobes	High sensitivity	Radionuclides must be used

**Table 2:** Studies investigating applications of single-modality nanoprobes in atherosclerosis research

Imaging properties	Nanoprobes	Models	Targets	Mechanisms	Applications	References
Optical imaging nanoprobes	Aggregation-induced emission nanoprobes	*Apoe^-/-^* mice	CD47	Regulation of the substituent of rhodanine	Early detection of plaques and screening of anti-AS drugs	28
	Composite mesoporous silica nanoparticles	*Apoe^-/-^* mice; RAW264.7 cells	CD36	Targeting macrophages	Inhibition and imaging of plaques	69
	β-galactosidase-activatable nanoprobes	*Apoe^-/-^* mice	β-galactosidase	Targeting senescent vascular cells	Early diagnosis and therapy of AS	66
	Luminescence nanoprobes	*Apoe^-/-^* mice	CD36 activation, CD36-oxLDL binding	Targeting foam cell formation	Monitoring the progression of atherogenesis	61
	MMP-2 aptamer-conjugated fluorescent nanoprobes	*Apoe^-/-^* mice	MMP-2	Targeting MMP-2 protein	Diagnostic tools of AS and cancer	65
Photoacoustic imaging nanoprobes	TiO_2_ nanoprobes	RAW 264.7 cells	Intracellular lipids	Cholesterol regulation pathways	Mild phototherapy	29
	Semiconducting polymer nanoparticle-based probes	*Apoe^-/-^* mice	CD36	Targeting inflammation of carotid plaques	Non-invasive imaging and assessment of plaques	80
	OPN Ab/Ti3C2/ICG nanoprobes	*Apoe^-/-^* mice	Osteopontin	Targeting VASPs	Differentiation of VASPs	79
	BSA-Cy-Mito nanoprobes	*Apoe^-/-^* mice	BSA	Targeting ox-LDL-activated macrophages	Early identification of rupture-prone plaques	78
	ICG@PEG-Ag_2_S photoacoustic nanoprobes	*Apoe^-/-^* mice	C18/PEG polymer molecules	Due to the lipophilicity of the C18 chain to AS microenvironment	Imaging of plaques	76
	AuNRs-Abs	Atherosclerotic rabbits; HUVECs	MMP-2	Targeting inflammation	Imaging of plaques	77
MR imaging nanoprobes	Fe_3_O_4_@M nanoparticles	Wistar rats	VCAM-1	The specific recognition of integrin α4β1 to VCAM-1	Diagnosis of early stage plaques	88
	Anti-IL-6-USPIO	Atherosclerotic rabbits; HUVECs	IL-6	Targeting inflammatory cytokines	Imaging of VASPs	87
	Tissue factor-targeting magnetic nanoprobes	*Apoe^-/-^* mice	Tissue factor	Targeting TF-positive atherosclerotic plaques	Detection of plaques	86
	Hyaluronan-conjugated magnetic nanoworms	*Apoe^-/-^* mice	CD44	Targeting CD44-expressing cells in plaques	Detection of plaques	92
	Sulfated dextran-coated iron oxide nanoprobes	J774 macrophages	SR-A	Targeting macrophages	Imaging of VASPs	95
	Gadolinium immunonanoparticle-based nanoprobes	*Apoe^-/-^* mice	MRP 8/14 complex	Targeting inflammation	Potential therapy of atherosclerosis	91
PET imaging nanoprobes	25% ^64^Cu-CANF-comb	C57BL/6 mice*; Apoe^-/-^* mice	NPR-C	Targeting NPR-C expression	Detection of plaques	103
	^64^Cu-DOTA-CANF-comb nanoprobes	Murine HLI Model	NPR-C receptors	Targeting angiogenesis	Imaging NPR-C receptor in angiogenesis	100, 101
	^89^Zr-discoidal HDL nanoparticles	*Apoe^-/-^* mice; rabbits; pigs	HDL	Targeting plaque macrophages and monocytes	Imaging of plaques	102

**Table 3:** Studies investigating applications of dual-modality nanoprobes in atherosclerosis research

Imaging properties	Nanoprobes	Models	Targets	Mechanisms	Applications	References
Optical/MR imaging nanoprobes	TPZ/IR780@ HSAeOPN nanoprobes	*Apoe^-/-^* mice	Osteopontin	Targeting VASPs	Identification of VASPs and regression of plaques	27
	VEGFR2-targeted upconversion nanoprobes	*Apoe^-/-^* mice; HUVECs	VEGFR2	Targeting angiogenesis	Imaging of VASPs	119
	ROS-Scavenging Nanoparticles	*Apoe^-/-^* mice; RAW264.7 cells	ROS scavengers	Targeting macrophages	Imaging and anti-ROS treatment of VASPs	48
	MARCO-targeted upconversion luminescence probes	*Apoe^-/-^* mice; BMMCs	MARCO	Targeting M1 macrophage polarization	Imaging of VASPs	46
	PP1-Au@GSH@Gd NCs	*Apoe^-/-^* mice; RAW264.7 cells	SR-AI	Targeting foam macrophages	Imaging of VASPs	118
	Fe_3_O_4_ nanoparticle-based probes	*Apoe^-/-^* mice	Osteopontin	Targeting foamy macrophages	Imaging of VASPs	111
	OPN-specific upconversion luminescent probes	*Apoe^-/-^* mice; Raw 264.7 cells	Osteopontin	Targeting foamy macrophages	Imaging of VASPs	114
	PC-NPs	*Apoe^-/-^* mice; MOVAS	Profilin-1	Targeting VSMCs	Detection of plaques	120
	Fluorescent iron oxide magnetic nanoprobes	*Apoe^-/-^* mice; RAW264.7 cells	Folate receptor	Targeting activated macrophages	Detection of FRβ-enriched inflammatory plaques	45
Optical/ ultrasound imaging nanoprobes	COP-NP-based nanoprobes	*Apoe^-/-^* mice	Osteopontin	Targeting VASPs	Imaging of VSMCs and foam cells	123
Photoacoustic/ ultrasound imaging nanoprobes	RGDfk peptide-targeted BSA-based nanoprobes	Rabbits	RGDfk peptide	Targeting new blood vessels in vulnerable plaques	Visualization of VASPs	47
	Silica-coated gold nanorods	J774A.1; patients	-	Targeting activated macrophages	Detection and temperature monitoring plaques	126
PET/CT imaging nanoprobes	^64^Cu-DAPTA-comb	*Apoe^-/-^* mice	CCR5	Characterizing CCR5 expression	Imaging the progression and regression of plaques	132
	^64^Cu-vMIP-II-comb	*Apoe^-/-^* mice	Chemokine receptors	Detection of chemokine receptors	Assessment of plaque progression	131
	^64^Cu-DOTA-DAPTA-comb	C57BL/6 mice; *Apoe^-/-^* mice	CCR5	Targeting CCR5	Imaging CCR5 expression in plaques	130
PET/MR imaging nanoprobes	^64^Cu-MMP2cNPs	CL57/BL6 mice	MMP-2	Targeting macrophages	Detection of MMP-2 in plaques	137
	^68^Ga-HAP-multitag nanoprobes	*Apoe^-/-^* mice	HAP	Characterizing vascular calcifications in plaques	Imaging of VASPs	97
	^68^Ga-iron oxide nano-radiomaterials	*Apoe^-/-^* mice	OxLDL	Targeting oxidized phospholipids	Detection of plaques	139
	^68^Ga-NGD-MNPs	Rabbits	GEBP11 Peptide	Targeting angiogenesis	Imaging of VASPs	138
	^64^Cu-dextran sulfate coated iron oxide nanoparticles	SD rats; *Apoe^-/-^* mice	Macrophages	Targeting vascular inflammation	Identification of vulnerable plaques	136

**Table 4:** Several well-investigated molecular targets in non-invasive imaging for atherosclerotic plaques

Main processes	Molecular targets	Expression	Main biological functions	Formulated nanoprobes
Targeting inflammation	VCAM-1	Immune cells and vascular endothelium	Cell adhesion	MR imaging nanoprobes
	Osteopontin	Macrophages	Biomineralization, cell adhesion	Photoacoustic, MR imaging nanoprobes
	IL-6	Immune cells, smooth muscle cells, endothelial cells, adipocytes	Acute phase	MR imaging nanoprobes
	MMP-2	Fibroblasts	Angiogenesis, collagen degradation	Optical, photoacoustic imaging nanoprobes
	CD36	Monocytes, endothelial cells, adipocytes, skeletal and cardiac muscle cells	Cell adhesion, lipid transport	Optical, photoacoustic imaging nanoprobes
	SR-AI	Macrophages, Hepatocytes, Adipocytes, Kupffer cells, Granulosa cells	Host-virus interaction	MR imaging nanoprobe
	CD47	Monocytes/macrophages	Cell adhesion	Optical imaging nanoprobes
	MARCO	Macrophages, Kupffer cells	Immunity, innate immunity	Optical/MR imaging nanoprobes
Targeting angiogenesis	VEGFR2	HUVECs	Angiogenesis	Optical/MR imaging nanoprobes
	NPR-C receptors	The endothelium of neovessels	Angiogenesis	PET imaging nanoprobes
Targeting vascular calcifications	CD44	Myeloid cells	Cell adhesion and migration	MR imaging nanoprobes
Targeting lipoproteins	HDL	Plasma	Reverse cholesterol transport	PET imaging nanoprobes

## Abbreviations

CT: computed tomography
CTA: computed tomography angiography
MRI: magnetic resonance imaging
PET: positron emission tomography
AIE: aggregation induced emission
TNF: tumor necrosis factor
CD47: cluster of differentiation 47
PAI: photoacoustic imaging
IVPA: intravascular photoacoustic
IVUS: intravascular ultrasound
VASP: vulnerable atherosclerotic plaques
OPN: osteopontin
BSA: bovine serum albumin
SPIONs: superparamagnetic iron oxide nanoparticles
TF: tissue factor
CD36: cluster of differentiation 36
LDL: low-density lipoprotein
oxLDL: oxidized low-density lipoprotein
MRP: Myeloid-related protein
HLI: hind limb ischemia
CANF: ^64^Cu-labeled C-type atrial natriuretic factor
MMP-2: matrix metalloproteinase-2
ICG: indocyanine green
NPR-C: natriuretic peptide clearance receptor
COP-NPs: Cy5.5-anti-OPN-PEG-PLA-PFOB nanoparticles
VSMCs: vascular smooth muscle cells
MARCO: macrophage receptor with collagenous structure
BMMCs: bone marrow mononuclear cells
SR-A: scavenger receptor A
VEGFR2: vascular endothelial growth factor receptor 2
ROS: reactive oxygen species
HDL: high-density lipoprotein
AuNRs-Abs: gold nanorods conjugated with MMP-2 antibody
IL-6: interleukin-6
Anti-IL-6-USPIO: IL-6-targeted superparamagnetic iron oxide nanoparticles
^18^F-FDG: (18)F-fluorodeoxyglucose
NIR: near-infrared
FR: folate receptor
HA: hyaluronan
HAP: hydroxyapatite
MMP2cNPs: ^64^Cu-NOTA-IONP@MMP2c-PEG2K
VCAM-1: vascular cellular adhesion molecule-1
